# HC-Net: A hybrid convolutional network for non-human primate brain extraction

**DOI:** 10.3389/fncom.2023.1113381

**Published:** 2023-02-09

**Authors:** Hong Fei, Qianshan Wang, Fangxin Shang, Wenyi Xu, Xiaofeng Chen, Yifei Chen, Haifang Li

**Affiliations:** ^1^College of Information and Computer, Taiyuan University of Technology, Taiyuan, China; ^2^Country Intelligent Healthcare Unit, Baidu, Beijing, China

**Keywords:** brain extraction, deep learning, hybrid convolution network, hybrid features, non-human primate MRI

## Abstract

Brain extraction (skull stripping) is an essential step in the magnetic resonance imaging (MRI) analysis of brain sciences. However, most of the current brain extraction methods that achieve satisfactory results for human brains are often challenged by non-human primate brains. Due to the small sample characteristics and the nature of thick-slice scanning of macaque MRI data, traditional deep convolutional neural networks (DCNNs) are unable to obtain excellent results. To overcome this challenge, this study proposed a symmetrical end-to-end trainable hybrid convolutional neural network (HC-Net). It makes full use of the spatial information between adjacent slices of the MRI image sequence and combines three consecutive slices from three axes for 3D convolutions, which reduces the calculation consumption and promotes accuracy. The HC-Net consists of encoding and decoding structures of 3D convolutions and 2D convolutions in series. The effective use of 2D convolutions and 3D convolutions relieves the underfitting of 2D convolutions to spatial features and the overfitting of 3D convolutions to small samples. After evaluating macaque brain data from different sites, the results showed that HC-Net performed better in inference time (approximately 13 s per volume) and accuracy (mean Dice coefficient reached 95.46%). The HC-Net model also had good generalization ability and stability in different modes of brain extraction tasks.

## 1. Introduction

With the launch of brain science programs in various countries, macaques have become an important non-human primate animal model (Zhang and Shi, 1993; Li et al., 2019). Researchers have conducted invasive experiments (such as electrophoresis, biology, histology, and lesions) on macaque brains to verify hypotheses that cannot be carried out on human brains (Liu et al., 2018; Cai et al., 2020). In brain science research, MRI has become an essential medical technology to study the brain because of its non-invasiveness, ease of collecting information many times, and rich and detailed tissue information. Brain extraction is one of the initial steps of MRI image processing (Esteban et al., 2019; Tasserie et al., 2020). By removing non-brain tissues (skull, muscle, eye, dura mater, external blood vessels, and nerves), the accuracy of brain image processing steps can be improved, such as anatomy-based brain registration, meningeal surface reconstruction, brain volume measurement, and tissue recognition (Xi et al., 2019a,b; Autio et al., 2020; Lepage et al., 2021). However, the performance of existing brain extraction tools is lacking when applied to the macaque brain (Zhao et al., 2018).

**FIGURE 1:**
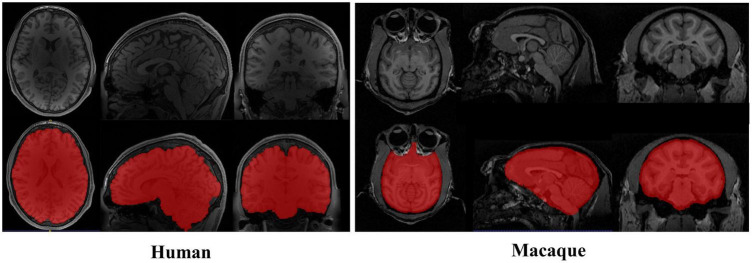
Examples of brain magnetic resonance imaging (MRI) images showing the tissue structure different between human and macaque brain. The red regions denote the brain. Compared with human brain, the macaque has narrow and prominent frontal lobe and eyes surrounded by adipose tissue.

The particularity of the macaque’s brain makes brain extraction more challenging than in humans. It mainly includes the following aspects: (1) The evolutionary distance of 25 million years makes the brain weight of macaques approximately one-tenth that of humans (Nei et al., 2001; Donahue et al., 2016). The narrow and prominent frontal lobe and the eyes surrounded by fatty tissue near the brain of macaques all make it difficult to extract with methods based on humans, as shown in Figure 1. (2) The species differences between macaques and humans necessitate increased spatial resolution to achieve the anatomical resolution that can be compared between them. However, a smaller voxel means a lower signal-to-noise ratio. To improve the signal-to-noise ratio, researchers have collected macaque data under higher field strengths (such as 4.7 T, 7 T, 9.4 T, and 11.7 T). The ultrahigh field strength will increase the heterogeneity of B0 and B1, which will strongly influence the tissue contrast, thus reducing the data quality (Van de Moortele et al., 2005). (3) Different macaque data collection sites use specific collection protocols and equipment (Milham et al., 2018), resulting in significant differences in the data quality and characteristics. To solve these challenges, other researchers have proposed methods for non-human primate data [i.e., a new option “-monkey” in AFNI (Cox, 1996), registration methods (Lohmeier et al., 2019; Jung et al., 2021)]. However, the final results mostly require manual intervention. Therefore, an automatic, rapid, and robust brain extraction method for macaques is highly desirable in non-human primate studies.

In recent years, following the revival of deep neural networks (Seyedhosseini et al., 2013; Yan et al., 2015) and the development of parallel computing (Coates et al., 2013; Schmidhuber, 2015), DCNNs have shown excellent performance in various computer vision tasks and have been widely used in medical image segmentation for human body tissue (Zhao et al., 2018; Wang et al., 2021, 2022; Huang et al., 2022; Sun et al., 2022). The methods based on DCNN architecture can be divided into 2D (2D convolution kernel) and 3D (3D convolution kernel) methods. Based on the excellent feature extraction ability of the 2D convolution kernel, the 2D methods can extract features quickly from original volumes. However, the input of a 2D convolution network is usually a slice cut along the Z-axis, which ignores the spatial information of volume data. This would lead to limiting the ability of model segmentation. To overcome this limitation, 2D methods that make use of information from adjacent slices have been introduced (Lucena et al., 2019; Zhang et al., 2019). Specifically, adjacent slices cropped from volumetric images are fed into the 2D networks as the 3D segmentation volume by simply stacking the 2D slices. Although adjacent slices are employed, it is still not enough to probe the spatial information along the third dimension, which leads to the underfitting of 2D convolution to spatial information. In particular, macaque brain samples have the nature of thick-slice scanning, and more voxels are primarily anisotropic (0.60×1.20×0.60 mm, 0.50×0.55×0.55 mm, 0.75×0.75×0.75 mm), which corrupts the extraction of brain tissue from macaque brain MRI images. To make full use of the context information of 3D medical volume data, 3D DCNNs are applied to the field of brain image segmentation (Hwang et al., 2019; Coupeau et al., 2022). Kleesiek et al. (2016) first proposed an end-to-end 3D DCNN for human brain extraction, and then 2D U-Net was also extended to 3D for the 3D dataset (Çiçek et al., 2016). Milletari et al. (2016) used 3D FCN and dice loss to construct a v-net network for MR image segmentation, and then Dolz et al. (2018) used it to segment subcutaneous brain tissue. Compared with 2D networks, 3D networks suffer from high computational costs and GPU memory consumption. The high memory consumption limits the depth of the network as well as the filter’s field of view. To take advantage of 3D information and reduce the negative impact of 3D networks, Li et al. (2018) used a 2D H-DenseUNet to extract the features within the slices and a 3D H-DenseUNet to extract the features between the slices. Furthermore, the work formulated the learning process in an end-to-end manner. The intra-slice representations and inter-slice features were fused by the HFF layer, which improved the segmentation effect of the liver and tumor. In human brain data, hundreds of training samples and validation samples are used to ensure accuracy and relieve the overfitting of the model. However, compared with the human brain, the small sample characteristics of macaques limit the training of an additional 3D network. On the other hand, Prasoon et al. (2013) sliced on the X, Y, and Z axes and input the slices of the different axes into three 2D FCNs to compensate for the absence of the 3D features in training. Similarly, to reduce training time, Chen et al. (2021) proposed a triple U-Net composed of three U-Net networks, and the input of three networks is one frame slice image (each frame of slice data contains three adjacent slices). Two auxiliary U-Net networks supplemented and constrained the training of the main U-Net network, which significantly improved the accuracy of whole-brain segmentation. These studies have shown that the applications of intra-slice representations and inter-slice features are more conducive to improving accuracy in medical image segmentation. However, using multiple networks for feature fusion will increase the complexity and computational cost of the network. Therefore, a separate network to fuse 2D and 3D information to reduce the amount of calculation and simplify the training process for macaque brain extraction requires further research.

The present work attempts to overcome the above problems to develop a general brain extraction model based on deep learning for non-human primates. To achieve higher accuracy, the model can efficiently extract the intra-slice representations and inter-slice features from insufficient macaque data. It can also have better generalization and stability on untrained data sites. The main contributions of our research are threefold. First, to overcome the challenge of small sample sizes, the present work increases the amount of data by using slices in three directions of volume data. Second, our research uses 3D convolutions to extract the brain directly from the “Data Block”, which effectively represents more spatial features and relieves the underfitting of 2D convolutions to spatial features. Compared with directly loading 3D volume data, this method reduces the amount of computation. Finally, this research proposes an end-to-end hybrid convolutional encoding and decoding structure model (HC-Net) to balance the calculation and performance. The model represents the spatial information of existing data better and achieves higher extraction accuracy. Besides, the model reduces the learning burdens and the training complexity.

## 2. Materials and methods

### 2.1. Dataset

The MRI macaque data are publicly available from the recent NHP data-sharing consortium - the non-human PRIMate Data Exchange (PRIME-DE) (Milham et al., 2018). This research selected one anatomical T1w image per macaque in our study. Because the number of samples of individual data site is too small, we used the joint data of multiple sites [Newcastle University Medical School (Newcastle), *N* = 5, the University of California, Davis (ucdavis), *N* = 5, Mount Sinai School of Medicine (Phillips) [Mountsinai-P], *N* = 5, Stem Cell and Brain Research Institute (sbri), *N* = 5, University of Minnesota (UMN), *N* = 2, Institute of Neuroscience (Ion), *N* = 5, East China Normal University Chen (ecnu-chen), *N* = 5] for training and testing (Macaque dataset I, *N* = 32). The data of different field strengths [Lyon Neuroscience Research Center (Lyon), 1.5 T, *N* = 4; Mount Sinai School of Medicine Siemens scanner (mountsinai-S), 3 T, _*N=5*_; Newcastle University Medical School (Newcastle), 4.7 T, *N = 5*; University of Minnesota (UMN), 7 T, *N* = 2; University of Western Ontario (UWO), 7 T, *N* = 3] were used as an additional dataset (Macaque dataset II, *N* = 19) to further verify the performance of the model. Detailed data information about the data alliance can be found on the website https://fcon_1000.projects.nitrc.org/indi/indiPRIME.html. Each selected T1w image was segmented manually to make a ground truth mask.

Human T1w MRI images and macaque B0 images were used to extend the proposed model to facilitate the brain extraction of different species and modes. The human data used in the present study are publicly available from the Human Connectome Project (HCP) (Van Essen et al., 2012), WU-Minn 1,200 subjects data release. In this study, the training dataset included 50 brain T1w subjects, and the test dataset included 17 subjects. The ground truth masks were created by their corresponding brain tissue files. The macaque B0 images were obtained from diffusion-weighted imaging (DWI) data in the UWM dataset of PRIME-DE. To improve the image quality, head motion eddy current correction and gradient direction correction were carried out for the original DWI images. In particular, to reduce the workload of manual brain extraction, this work applied the existing T1w image mask registration to B0 images to eliminate most non-brain tissue. At this time, the B0 images still contain non-brain tissues such as eyeballs and fat. The ground truth masks were manually made for the B0 images. The samples of 25 macaques were used for training, and 10 were tested.

### 2.2. “Data Block” pre-processing

**FIGURE 2:**
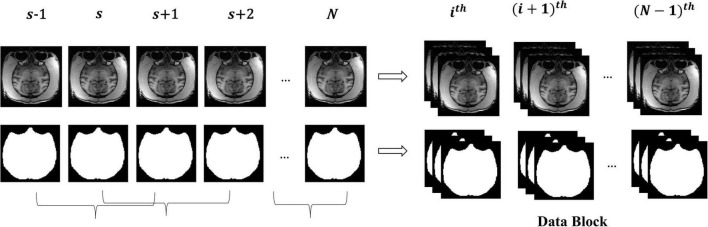
The manufacturing process of the “Data Block”.

The macaque MRI image is a three-dimensional volume, and there is context information between consecutive slices. For training, the slices will be independently input into the model, which will destroy the dependency between slices. Therefore, this research took three consecutive slices as a “Data Block” to maintain this relationship between slices and smooth the contour of brain tissue by constraints from adjacent slices. Figure 2 shows the manufacturing process of the “Data Block”. We continuously read three adjacent 2D slices *s*−1,*s*,*s* + 1 (*s* ∈ [2,*N*−1], where _*N*_ is the number of sample slices) as a “Data Block” in gray mode. This research took the middle slice label of this block as the label. The reading step was set to 1, that was, the *i^th^* block was (*s*−1,*s*,*s* + 1), and the block (*i* + 1)^*th*^ was (*s*,*s* + 1,*s* + 2).

To increase the training data, each sample was sliced along the coronal plane, sagittal plane, and horizontal plane. The final dataset was obtained by splicing slices of three planes. The initial T1w data are anisotropic. To merge the probability maps of the three axes, we resampled the image to a size of 256×256 by using the double trilinear interpolation method. Furthermore, to reduce the heterogeneity between the data of different sites and improve the quality of images, the intensity of the data was standardized so that the intensity values were between 0 and 1. T1w MRI images of humans and B0 images of macaques were also pre-processed in this way.

### 2.3. HC-Net network

Although 2D convolutions have achieved great success in many segmentation tasks, they are incapable of exploring inter-slice information. To this end, this study first uses two 3D convolution blocks to obtain more context information of slices from the original volumes. Then, to reduce the amount of computation and increase the receptive field of the network, the proposed model employs the encoding and decoding structure to construct the HC-Net network, which can be trained with a small dataset. Finally, the skip connection retains the details after each encoding to reduce the loss of bottom features and integrate multiscale information to improve performance.

**FIGURE 3:**
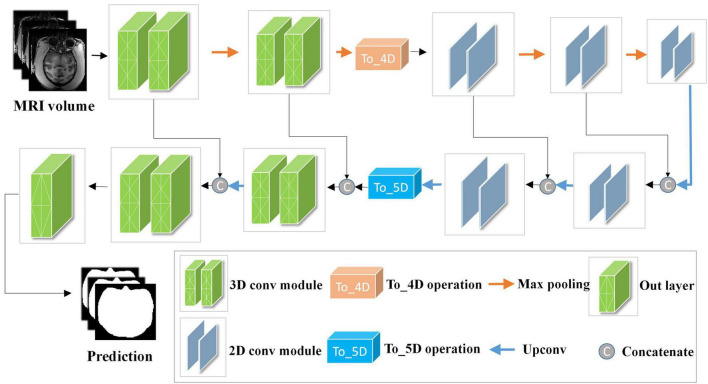
Architecture of the hybrid convolutional neural network (HC-Net). Given the input “Data Block”, two 3D encoders are first used to obtain more context information of slices. Then, 2D encoders are utilized to reduce the amount of computation. Finally, the encoding and decoding structure are used to increase the receptive field of the network and to restore the size of the original image.

The network structure of HC-Net is shown in Figure 3. It includes the encoding path and decoding path. The encoding path comprises five encoders, and each encoder is composed of two convolution layers. The first and second encoders are 3D convolution modules connected with a normalization operation and a ReLU activation function after convolution. With the 3D convolution kernels, these encoders can better extract spatial information from the original volumes. The third to fifth encoders are 2D convolution modules. They can significantly reduce the number of parameters and complexity of model training. To achieve this, the *To*_4*D* operation is used to convert tensors of different dimensions. A max-pooling operation is used after each encoder to reduce the image resolution in the encoding path. In the decoding path, there are four decoders. The first two decoders are 2D modules, and the last two decoders are 3D modules. Each decoder is connected with a permutation convolution and a ReLU activation function for upsampling. After upsampling, each decoder concatenates the feature maps of the corresponding size in the encoding path by the skip connection. After upsampling three times, through the *To*_5*D* operation, the feature maps are converted into 5-dimensional tensors and concatenated with the corresponding feature maps in the encoding path. Then, the hybrid feature maps are input into 3D decoders. After the last decoder, this study employs a 1×1×1 convolution layer to map the final feature maps to a two-class map. Finally, a softmax layer is used to obtain the probability map for the brain tissue.

**FIGURE 4:**
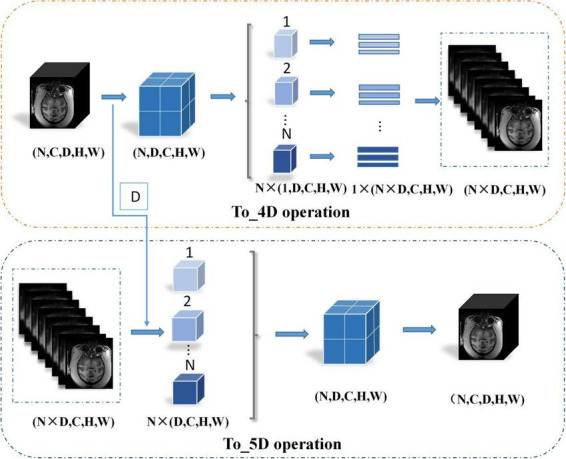
Details of the To_4D operation and To_5D operation.

We denote the input training samples as *IN* ∈ *R*^*N*×*C*×*D*×*H*×*W*^ with ground truth labels *G* ∈ *R*^*N*×*C*×*D*×*H*×*W*^, where *N* denotes the batch size of the input training samples, *C* denotes the channel, and _*D×H×W*_ denotes the size of the samples. *G*(*x*,*y*,*z*) = 0 or 1 indicates that the pixel (*x*,*y*,*z*) is tagged with the class brain (1) or non-brain (0). Let *IN*_3d_ ∈ *R*^*N*×1×3×256×256^ denote the input of the first encoder and *F*_3d_ denote some sequence operations of 3D convolution, batch normalization, and the activation function. The learning process of 3D convolutions in the encoding path can be described as follows:


(1) $${\text{X}}_{\mathrm{e2}}=F_{3\,d}({\text{IN}}_{3d)},{\text{X}}_{\mathrm{e2}}\in {\text{R}}^{N\times 32\times 3\times 64\times 64}$$

where *X*_*e2*_ represents the features after the second encoder. The *To*_4*D* operation converts the 5-dimensional tensors into 4-dimensional tensors by stacking the batch and depth dimensions and inputting them into the 2D encoder. First, this research records the depth dimension and then swaps the channel dimension and depth dimension. Second, the data are split along the batch size dimension and then spliced along the depth dimension. Finally, the tensor is compressed into a 4-dimensional tensor. The details of the *To*_4*D* operation are shown in Figure 4. The *To*_4*D* operation is as follows:


(2) $${\text{X}}_{\mathrm{e2}}^{{}^{\prime }}=To\_4D({\text{X}}_{\mathrm{e2})},{\text{X}}_{\mathrm{e2}}^{{}^{\prime }}\in {\text{R}}^{3\,N\times 32\times 64\times 64}$$

where ${\text{X}}_{\mathrm{e2}}^{{}^{\prime }}$ denotes the input data of the third encoder.

*F*_2*d*_ denotes some sequence operations of the 2D convolution, batch normalization, and activation functions, and the training processing of the 2D encoder and decoder can be denoted as:


(3) $${\text{X}}_{\mathrm{d3}}=F_{2\,d}\,\left({\text{X}}_{\mathrm{e2}}^{{}^{\prime }}\right),{\text{X}}_{\mathrm{d3}}\in {\text{R}}^{3\,N\times 32\times 128\times 128}$$

where **X_d3_** denotes the feature maps from the third upsampling layer. The 4D tensor is converted into the 5D tensor by splitting the batch size dimension to restore the depth dimension,


(4) $${\text{X}}_{\mathrm{d3}}^{{}^{\prime }}=T\,o\,\_\,5\,D\,\left({\text{X}}_{\mathrm{d3}},D\right),{\text{X}}_{\mathrm{d3}}^{{}^{\prime }}\in {\text{R}}^{N\times 32\times 3\times 128\times 128}$$

where *D* denotes the depth dimension from the *To*_4*D* operation and ${\text{X}}_{\mathrm{d3}}^{{}^{\prime }}$ denotes the 5D tensor feature volume from the third upsampling layer. The details of the *To*_5*D* operation are shown in Figure 4. Especially after 2D convolution in the decoding path, the 3D decoder is trained based not only on the features detected in the 2D decoder but also on the 3D context features from the second encoder. The hybrid features from the 2D and 3D convolutions are jointly learned after the third upsampling layer in the decoding path. The hybrid operation can be described as follows:


(5) $${\text{X}}_{h}={\text{X}}_{\mathrm{d3}}^{{}^{\prime }}+{\text{X}}_{{\mathrm{e2}}^{\prime }},{\text{X}}_{h}\in R^{N\times 64\times 3\times 128\times 128},X_{{\mathrm{e2}}^{\prime }}\in R^{N\times 32\times 3\times 128\times 128}$$

Where *X*_*h*_ denotes the hybrid features and *X*_*e*2′_ denotes the output of the second encoder without max-pooling. More details of the HC-Net network are given in Table 1.

**TABLE 1:** Architectures of the proposed HC-Net. The feature size column indicates the output size of the current stage. The “(3*D*,3×3×3,1,1)×2” corresponds to the 3D convolution with two convolution kernels of 3×3×3, stride 1 and padding 1. “MaxPool3d, 1×2×2,1×2×2” corresponds to max-pooling with a sliding window size of 1×2×2, stride 1×2×2. “2*DCT*,4×4,1,1” corresponds to 2D transpose convolution with a kernel of 4×4, stride 1, and padding 1.

	HC-Net	Feature size		HC-Net	Feature size
Input	−	3×256×256	Encode 1	(2*D*,3×3,1,1)×2	128×32×32
*Encoder1*	(3*D*,3×3×3,1,1)×2	16×3×256×256	Upconv	2*DCT*,4×4,2,1	64×64×64
*MaxPool3d*	1×2×2,1×2×2	16×3×128×128	Concatenate	−	128×64×64
*Encoder2*	(3*D*,3×3×3,1,1)×2	32×3×128×128	Decoder 2	(2*D*,3×3,1,1)×2	64×64×64
*MaxPool3d*	1×2×2,1×2×2	32×3×64×64	Upconv	2*DCT*,4×4,2,1	32×128×128
*To*_4D	−	3×32×64×64	To_5D	−	32×3×128×128
*Encoder3*	(2*D*,3×3,1,1)×2	64×64×64	Concatenate	−	64×3×128×128
MaxPool2d	2×2	64×32×32	Decoder3	(3*D*,3×3×3,1,1)×2	32×3×128×128
Encoder 4	(2*D*,3×3,1,1)×2	128×32×32	Upconv	3*DCT*,3×4×4,2,1	16×3×128×128
MaxPool2d	2×2	128×16×16	Concatenate	−	32×3×256×256
Encoder 5	(2*D*,3×3,1,1)×2	256×16×16	Decoder 4	(3*D*,3×3×,1,1)×2(3*D*,3×3×3,1,1)×2	16×3×256×256
Upconv	2*DCT*,4×4,2,1	128×32×32	Out layer	3*D*,3×3×3,1,1	2×3×256×256
Concatenate	−	256×32×32	−	−	−

### 2.4. Loss function and evaluation indicators

The cross-entropy loss function was employed as the loss function in this study to train the networks, which can be described as:


(6) $$L\,o\,s\,s\,\left(y,\hat{y}\right)=\frac{1}{N}\,\sum_{i}-\left[y_{i}^{c}\,\log\left(p_{i}^{1}\right)+\left(1-y_{i}^{c}\right)\,\log\left(1-p_{i}^{1}\right)\right]$$

Where $y_{i}^{c}$ indicates the ground truth label for voxel *i* (brain or non-brain) and $p_{i}^{1}$ denotes the probability of voxel *i* belonging to the brain.

In this paper, true position (*TP*), true negative (*TN*), false positive (*FP*), and false negative (*FN*) were used to mark the comparison between the extraction result and ground truth. The Dice coefficient (*Dice*), sensitivity (*Sen*), specificity (*Spe*), and volumetric overlap error (*VOE*) are mainly used to evaluate the model’s performance in medical image segmentation. These evaluation indicators can be formulated as follows:


(7) $$D\,i\,c\,e=\frac{2\,T\,P}{2\,T\,P+F\,P+F\,N}$$

(8) $$S\,e\,n\,s\,i\,t\,i\,v\,i\,t\,y\,\left(S\,e\,n\right)=\frac{T\,P}{T\,P+F\,N}$$

(9) $$S\,p\,e\,c\,i\,f\,i\,c\,i\,t\,y\,\left(S\,p\,e\right)=\frac{T\,N}{F\,P+T\,N}$$

(10) $$V\,O\,E=1-\frac{T\,P}{F\,N+T\,P+F\,P}$$

The Dice coefficient is used to calculate the similarity between two samples whose value ranges between 0 and 1, and a higher value indicates similarity. This measurement function represents the ratio of the intersection area of two samples to the total area. Sensitivity is an index to measure the ability of the extraction algorithm to correctly identify the brain, which indicates the proportion of pixels correctly judged as brain tissue. Specificity is an index to measure the ability of the brain extraction algorithm to correctly identify non-brain tissue, indicating the proportion of pixels that are non-brain tissue that is correctly judged as non-brain tissue. The lower the volumetric overlap error is, the higher the sample similarity.

## 3. Experiments and results

### 3.1. Implementation details

The HC-Net model was implemented with the PyTorch framework and ran on an NVIDIA RTX 3090 GPU. It is trained end-to-end, which means that the “Data Block” is provided as input without any other process or additional network. The initial minimum learning rate was 1×10^−4^, the training batch size was 20, and the training epoch was 50. To train this network, this research employed the cross-entropy loss function to calculate the loss between the ground-truth labels and the predicted labels of the “Data Block”. The training time of the HC-Net model was approximately 4 h. For a fair comparison, state-of-the-art methods such as SegNet (Badrinarayanan et al., 2017), 2D U-Net (Ronneberger et al., 2015), 3D U-Net (Çiçek et al., 2016), U^2^-Net (Qin et al., 2020), and UNet 3+ (Huang et al., 2020) were trained with the same training data and tested on the same test data in all experiments.

### 3.2. Comparison with other methods

In this section, we conduct comprehensive experiments to analyze the effectiveness of our proposed method on dataset I.

**FIGURE 5:**
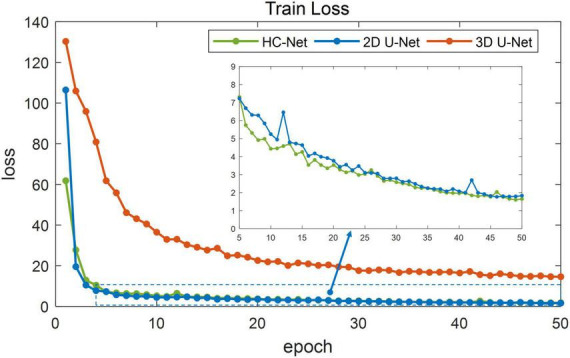
Loss in the training process of hybrid convolutional neural network (HC-Net), 2D U-Net, and 3D U-Net.

**FIGURE 6:**
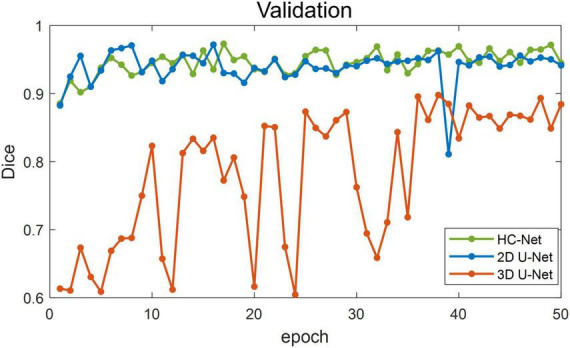
Dice coefficients on the validation set.

Figure 5 shows the training losses of 2D U-Net, 3D U-Net, and HC-Net. The loss converged faster for the HC-Net model than for the 2D U-Net and 3D U-Net models. In addition, the loss of the HC-Net model was the smallest. Figure 6 shows that the Dice coefficients are more stable for the HC-Net model in the validation dataset. As shown in Figures 5, 6, the performance of the 3D U-Net model was always lower than that of the 2D U-Net model under the same epoch (higher loss and lower Dice coefficients across 50 epochs). This highlighted the effectiveness and efficiency of 2D convolutions. This was because the 3D convolutions consume a large amount of GPU memory, so the network converged slowly. After the 40th epoch, the Dice coefficients of the validation set in the 3D U-Net model were relatively stable, but the values were lower than 0.9, and the expressiveness was weak. The HC-Net model showed a higher Dice coefficient, and the performance of the model tended to be stable. The result meant our model achieved better performance in macaque brain extraction.

The performances of different brain extraction methods were evaluated by using T1w images of 12 samples. These samples were not the participants for training and were located in different sites from the training sets. The extraction results from all test volumes were obtained and compared with the ground truth labels. Table 2 shows the average of the indicators. The HC-Net model outperformed the state-of-the-art methods by a margin on Dice. All the evaluation indicators of the HC-Net network were higher than those of FSL and AFNI. Compared with the Dice coefficient indicator, the proposed method was approximately 0.3% ∼10.48% higher than SegNet, 2D U-Net, 3D U-Net, U^2^-Net, and UNet3+. Experimental results confirmed that our model can robustly handle each example by incorporating the advantages of 3D convolution and 2D convolution for learning feature representations on intra-slice and inter-slice features.

**TABLE 2:** Evaluation results of different methods.

Method	Dice	Sen	Spe	VOE
FSL	0.7832	0.9372	0.9678	0.3251
AFNI	0.8471	0.8180	0.9928	0.2241
SegNet	0.9427	0.9652	0.9940	0.1079
2D U-Net	0.9454	0.9775	0.9929	0.1030
3D U-Net	0.8498	0.8234	0.9891	0.2197
U^2^-Net	0.9516	0.9787	0.9939	0.1068
UNet3+	0.9430	0.9775	0.9932	0.1121
HC-Net	0.9546	0.9464	0.9973	0.0860

Table 3 shows the parameters of each model and the average inference time per macaque brain for each network. Compared with 3D U-Net, SegNet, U^2^-Net, and UNet3+, the proposed model had fewer parameters and less average inference time. Although the next best performance in Table 2 was the U^2^-Net model, it had at least 8.13 times more parameters and requires 1.32 more inference time than the HC-Net network. With fewer parameters, the HC-Net model had an advantage in computational efficiency with the added advantage of being trainable with a smaller dataset without compromising performance. The parameters and inference time were not inferior to those of the 2D U-Net model. This result was reasonable because of the 3D convolution in the HC-Net network. However, the accuracy of HC-Net was higher than that of the 2D U-Net model. It also confirmed that adding context information to our HC-Net model was effective for the extraction of macaque brains.

**TABLE 3:** Comparison of weights and testing times of different methods on the test dataset.

Model	Weights (M)	Inference time (minute)
SegNet	24.9444	1.1573
2D U-Net	2.4666	0.1120
3D U-Net	8.0826	0.5436
U^2^-Net	44.0237	0.3020
UNet 3+	26.9747	6.6886
HC-Net	5.4117	0.2273

**FIGURE 7:**
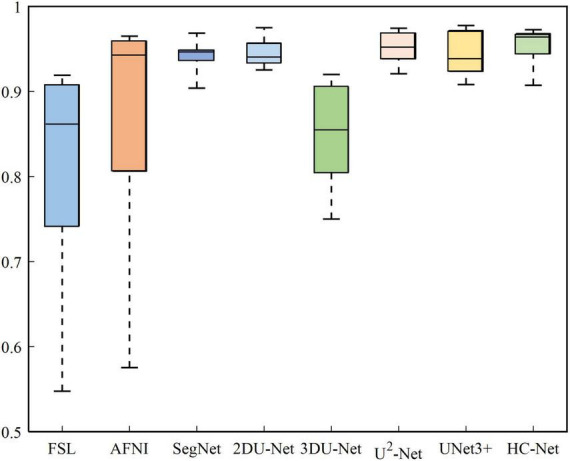
Box diagram of different methods.

**FIGURE 8:**
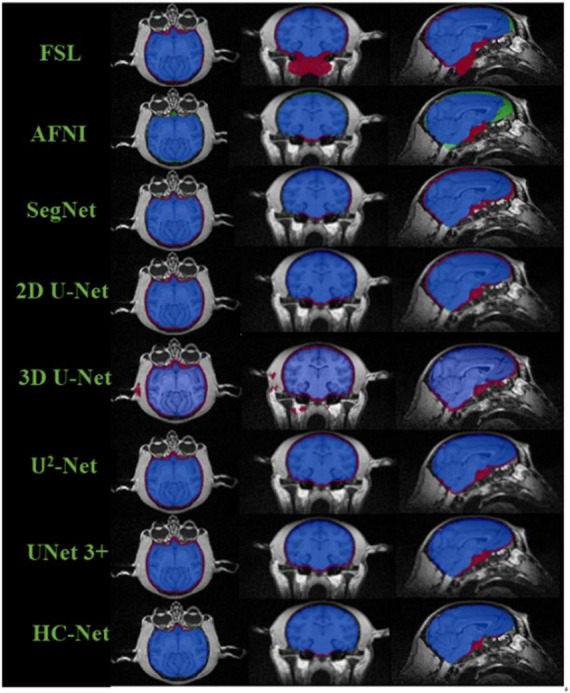
Comparison of segmentation results of different methods. The blue areas mean true positive (TP); the red areas mean false positive (FP); the green areas mean false negative (FN); and the rest areas mean true negative (TN).

The box plot results shown in Figure 7 show that the median of HC-Net was the highest. There was no prominent oscillation of the Dice coefficient on the test dataset, which showed good generalization and stability. Furthermore, Figure 8 shows the sample results of the brain extraction obtained by the present study and other methods on dataset I. The blue areas represent the true positive, the red areas mean the false positive, and the green areas mean the false negative. Compared with FSL, AFNI performed better by using the 3dskullstrip (-monkey) command dedicated to brain extraction. AFNI tended to be conservative in brain extraction and showed lower sensitivity, while FSL had low specificity and retains too many non-brain voxels. The 3D U-Net recognized some skulls as the brain. The possible reason was that for low-quality and small sample macaque data, 3D convolution also introduced more noise when retaining more information, resulting in over segmentation. The HC-Net generated a relatively complete mask without extending into the skull and missing parts of the brain tissue.

### 3.3. Evaluating the “Data Block”

This section used different data loading methods to verify the effectiveness of the “Data Block” used in this paper by dataset I. Experiments were carried out on the HC-Net model and 2D U-Net model with similar parameters. The same hyper parameters were used in all experiments (learning rate, loss function, etc.). This research used three pre-processing methods to process the data. The first method was to slice along the Z-axis of volume data and input one slice (1A1S) to the 2D U-Net model. The second method was to slice along the Z-axis and input three adjacent slices into the HC-Net and 2D U-Net models (1A3S). To increase the dataset, the setting step was also set to 1, that is, (*s*−1,*s*,*s* + 1, (*s*,*s* + 1,*s* + 2), where “*s*” represents the slice number. The third method (3A3S) was the “Data Block” introduced in part 2, which adds slices of the X and Y axes based on the second method.

Table 4 shows the experimental results. By inputting three axes into the network, the amount of data was three times higher than that of a single axis. Compared with the first method, the second method increased the Dice coefficient in the 2D U-Net model by 4.09%. Compared with 2D U-Net, using the second method to input adjacent slices into the HC-Net model dramatically improved the extraction results of the test data, in which the Dice coefficient, sensitivity, and specificity were increased by 11.54, 5.98, and 3.47%, respectively, and the VOE was reduced by 16.42%. The results showed that the HC-Net model can extract more features from limited data and improve the extraction effect through the second method. It also proved that the data pre-processing method of inputting three-axis data into the HC-Net model and 2D U-Net model can improve the accuracy of brain extraction with the increase in the training data. At the same time, HC-Net had higher sensitivity to data with spatial features and can fully extract the corresponding features. Therefore, the data pre-processing method of “Data Block” was appropriate for the proposed model and further improved the performance.

**TABLE 4:** The results of different pre-processing methods.

Model	Data	Dice	Sen	Spe	VOE
2D U-Net	1A1S	0.7612	0.8335	0.9599	0.3494
2D U-Net	1A3S	0.8021	0.8555	0.9564	0.3135
HC-Net	1A3S	0.9175	0.9144	0.9911	0.1493
2D U-Net	3A3S	0.9454	0.9775	0.9929	0.1030
HC-Net	3A3S	0.9546	0.9464	0.9973	0.0860

### 3.4. Evaluating the performance of the model under data of different field strengths

**FIGURE 9:**
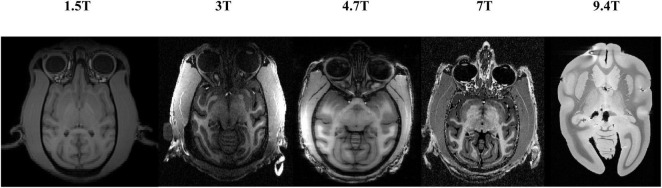
T1w images of macaques under different field strengths.

Significant differences in the brain signals captured under different field strengths (FS) are significant. Figure 9 shows the macaque data under 1.5 T, 3 T, 4.7 T, 7 T, and 9.4 T field strengths. The stronger the field strength is, the higher the signal-to-noise ratio. This means that the device can image at a higher or exact resolution, and the scanning speed is fast. The ultrahigh-field power will also increase the heterogeneity of B0 and B1, which will strongly influence the tissue contrast in the structure. To verify the generalization of the HC-Net model, macaque dataset II of different field strengths was used as the test dataset to further verify the model’s performance. Except for Newcastle (4.7 T), which had other data to participate in the training, other sites’ data had not participated in the training. The results of the 4.7 T data were for reference only.

Table 5 shows the brain extraction results on the HC-Net and 2D U-Net models. The Dice coefficient of the HC-Net model was approximately 0.89∼1.19% higher than that of the 2D U-Net. Importantly, our proposed model was better than the 2D U-Net model under different field intensities and had better generalization on data with different tissue contrasts.

**TABLE 5:** Results of different field strengths using the hybrid convolutional neural network (HC-Net) and 2D U-Net models.

Model	FS(T)	Dice	Sen	Spe	VOE
2D U-Net	1.5	0.9674	0.9970	0.9973	0.0629
HC-Net	1.5	0.9793	0.9788	0.9991	0.0405
2D U-Net	3	0.9058	0.9558	0.9933	0.1721
HC-Net	3	0.9125	0.8903	0.9975	0.1595
2D U-Net	4.7	0.9717	0.9813	0.9939	0.0548
HC-Net	4.7	0.9690	0.9488	0.9985	0.0179
2D U-Net	7	0.9415	0.9731	0.9920	0.1104
HC-Net	7	0.9504	0.9324	0.9976	0.0941

### 3.5. Evaluating the performance of the model on different datasets

Here, the human T1w MRI image dataset and the macaque B0 dataset were used to evaluate the utility of our proposed model. Table 6 shows the Dice coefficient, sensitivity, specificity, and VOE of the HC-Net model. The Dice coefficient, sensitivity, and specificity exceeded 98, 98, and 99%, respectively, in the two datasets, and the VOE was lower than 4%.

**TABLE 6:** The dice coefficient, sensitivity, specificity, and VOE of the hybrid convolutional neural network (HC-Net) model on the human dataset and B0 dataset.

Dataset	Dice	Sen	Spe	VOE
humans	0.9830	0.9868	0.9950	0.0332
B0 (macaques)	0.9841	0.9876	0.9989	0.0310

**FIGURE 10:**
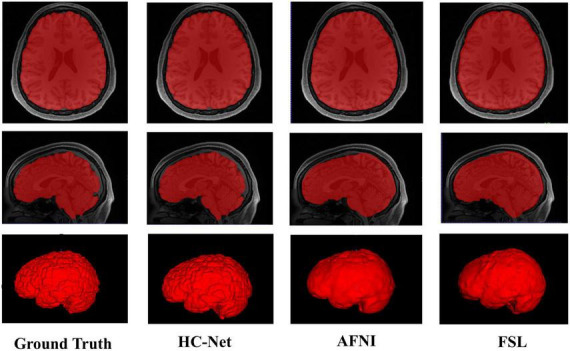
The results of human brain extraction using masks obtained by the ground truth, hybrid convolutional neural network (HC-Net model), AFNI, and FSL.

**FIGURE 11:**
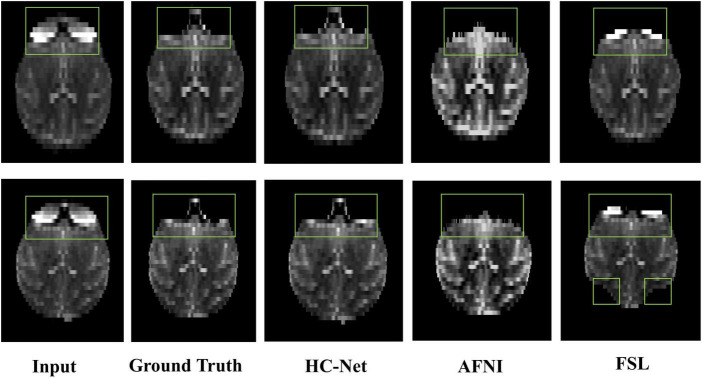
The results of macaques brain extraction using masks obtained by the ground truth, HC-Net model, AFNI, and FSL.

The proposed model had good stability in two datasets. Compared with macaques, the human cerebral cortex forms more folds on the surface of the brain. As such, folded and meandering brain morphology is one of the most difficult aspects of human brain extraction. Our HC-Net model correctly identified the boundary of the human brain, retained more details of the gyrus and sulcus, and obtained a more complete brain, as shown in Figure 10. AFNI and FSL smoothed the gyrus and sulcus excessively, resulting in loss of brain edge details. FSL failed when applied to the B0 image of macaques with eyes, as shown in Figure 11. It did not successfully separate the eyes. Furthermore, when no significant difference was present between the intensities of the brain and non-brain edges, it missed brain tissue. Compared with FSL, AFNI 3dSkullStrip with parameters customized for macaques showed better performance. However, AFNI 3dSkullStrip missed identifying the brain tissue around the eyes. Figure 11 shows two examples of the HC-Net model, and they performed outstandingly, showing little difference from ground truth masks.

## 4. Discussion

The present work demonstrated the feasibility of developing a brain extraction model of generalization and less training complexity for macaques by concatenating 3D and 2D convolutions. Central to the success of our effort was that this study fully extracted the spatial information between volume data by using 3D convolution and reduced the amount of calculation and parameters by 2D convolution. Our model overcame the problems of overfitting 3D convolution on small samples and underfitting 2D convolution on three-dimensional data. Compared with other cascade modes, our method reduced the complexity of training. Our work employed heterogeneous, multisite, different modes and different species of data resources to evaluate the effectiveness of the model. The results showed that the proposed model identifies the brains of macaques more accurately than the traditional methods. Furthermore, it had smaller parameters and better generalization than the large-scale model in small datasets. It was worth noting that our model also had excellent advantages in inference time.

Current works (Wang et al., 2021, 2022) show that macaque data samples for deep learning may not need to be as many as human data. The reason may be that the folding surface of the macaque brain is far less complex than that of humans (Hopkins et al., 2014), and the surface edge of macaque brain tissue is relatively smooth (Hopkins, 2018). At the same time, Croxson et al. (2018) showed that the similarity between individual macaques is higher than that between human samples, which also makes it possible for deep learning to train on small sample macaque data.

An essential discovery of the current work was the order of 2D convolution and 3D convolution. The research has proven the effectiveness of the serial convolution of 3D and then 2D for brain extraction. This study exchanged 2D and 3D convolution positions to build a new network. In the encoder stage, the network first used two 2D convolution modules and then three 3D convolution modules, and in the decoder stage, it first used two 3D convolution modules and then two 2D convolution modules. This new network reduced the training time and reasoning time because 3D convolution processing of large images required more computing power and time. However, the network was deficient in feature extraction after the exchange, which was even worse than that of the 2D U-Net model. The first reason may be that 3D convolution is more challenging to train in the middle of the model. The second reason may be that the spatial information that 3D convolution can learn from the feature map is limited when 3D convolution is trained after 2D convolution. In the HC-Net model, 3D convolution first extracts the spatial information of the original image. This spatial information is not only input into the 2D encoders but also fused with the information after decoding by skip connection. This method makes full use of spatial information.

It is worth noting that the HC-Net we proposed is not a complete 3D network, although the 3D network usually tends to have higher accuracy on large sample data. HC-Net has a smaller network scale, lower memory cost, and less computing time. In addition, HC-Net is easier to transplant on platforms with limited memory. The study used the data of three axes to increase the sample size and smooth the contour of the brain by constraints from adjacent slices. The experiment shows that this data enhancement method helps improve the model’s accuracy. However, there are differences between the data on the three axes of volume data. At the same time, to synthesize the final probability map, our study needs to resample the data, which loses some information. The introduction of data differences and the loss of information will affect the segmentation results to a certain extent. Future work may consider reducing the loss of information.

## 5. Conclusion

This research proposed an end-to-end trainable hybrid convolutional neural network, HC-Net, for brain extraction from MRI brain volumes of non-human primates. This study was a new way to extract inter-slice and intra-slice information from volume data by concatenating 2D and 3D convolutions. It reduced the calculation consumption and promotes accuracy by combining three consecutive slices from three axes for 3D convolutions. This architecture solved the problem that 2D convolution ignores the context information of volume data and the overfitting of 3D convolutions to small samples. Our model achieved excellent performance on limited macaque data samples. Experiments on human data and macaque B0 data also proved the effectiveness of our proposed HC-Net model.

## Data availability statement

The raw data supporting the conclusions of this article will be made available by the authors, without undue reservation.

## Author contributions

HL and QW evaluated and guided the experimental design of this research. HF conceived and designed the experiments and wrote the manuscript. FS and WX researched the data. XC and YC analyzed the results. All authors contributed to the article and approved the submitted version.
